# Reduced mRNA and Protein Expression of the Genomic Caretaker RAD9A in Primary Fibroblasts of Individuals with Childhood and Independent Second Cancer

**DOI:** 10.1371/journal.pone.0025750

**Published:** 2011-10-03

**Authors:** Eva Weis, Holger Schoen, Anja Victor, Claudia Spix, Marco Ludwig, Brigitte Schneider-Raetzke, Nicolai Kohlschmidt, Oliver Bartsch, Aslihan Gerhold-Ay, Nils Boehm, Franz Grus, Thomas Haaf, Danuta Galetzka

**Affiliations:** 1 Institute of Human Genetics, University Medical Center, Mainz, Germany; 2 Institute of Medical Biometry, Epidemiology and Informatics, University Medical Center, Mainz, Germany; 3 Experimental Ophthalmology, Ocular Proteomics and Immunology Center, University Medical Center, Mainz, Germany; 4 Institute of Human Genetics, Julius Maximilians University, Würzburg, Germany; Institut Jacques Monod, France

## Abstract

**Background:**

The etiology of secondary cancer in childhood cancer survivors is largely unclear. Exposure of normal somatic cells to radiation and/or chemotherapy can damage DNA and if not all DNA lesions are properly fixed, the mis-repair may lead to pathological consequences. It is plausible to assume that genetic differences, i.e. in the pathways responsible for cell cycle control and DNA repair, play a critical role in the development of secondary cancer.

**Methodology/Findings:**

To identify factors that may influence the susceptibility for second cancer formation, we recruited 20 individuals who survived a childhood malignancy and then developed a second cancer as well as 20 carefully matched control individuals with childhood malignancy but without a second cancer. By antibody microarrays, we screened primary fibroblasts of matched patients for differences in the amount of representative DNA repair-associated proteins. We found constitutively decreased levels of RAD9A and several other DNA repair proteins in two-cancer patients, compared to one-cancer patients. The RAD9A protein level increased in response to DNA damage, however to a lesser extent in the two-cancer patients. Quantification of mRNA expression by real-time RT PCR revealed lower *RAD9A* mRNA levels in both untreated and 1 Gy γ-irradiated cells of two-cancer patients.

**Conclusions/Significance:**

Collectively, our results support the idea that modulation of RAD9A and other cell cycle arrest and DNA repair proteins contribute to the risk of developing a second malignancy in childhood cancer patients.

## Introduction

In most cases, cancer is a multifactorial disease caused by environmental hazards, unhealthy lifestyle and/or genetic factors [1]. Because children are usually less exposed to an unfavourable environment or lifestyle than adults, genetic factors are likely to be a more important [2]. However, only a small proportion (1–10%) of childhood cancers has a known genetic etiology [3]. It is well known that irradiation and other DNA damaging agents used for cancer treatment are able to trigger the formation of leukemia and other cancers [4], [5]. Radiation and/or chemotherapy constitute risk factors for development of a second malignancy, which cannot be classified as remission of the primary tumor. Because relatively few childhood cancer survivors develop a second malignancy [6], genetic predisposition may be involved.

Cells are constantly exposed to endogenous and exogenous DNA damaging agents. There are several pathways that monitor and maintain genome integrity. Cells have multiple checkpoints that transiently delay cell cycle progression to allow extra time for DNA repair or induce apoptosis [7], [8]. Mutations or aberrant regulation of genes that control cell cycle checkpoints and DNA repair play important roles in tumorigenesis [9], [10] and are prime candidates when searching for genes modulating the risk for secondary cancer. If therapy-induced DNA damage is misrepaired, this can initiate second tumor development. Genetic predisposition may lead to increased chromosomal instability after radiation or chemotherapy [5], [11], [12]. Only in very rare cases of second childhood malignancy a genetic instability syndrome such as Fanconi anaemia, ataxia teleangiectasia or xeroderma pigmentosum has been diagnosed [10]. In most cases, the causes underlying development of a second cancer remain unknown.

To test the hypothesis that modulations in the expression of cell cycle control and DNA repair genes are associated with secondary cancer, we analyzed primary fibroblasts of childhood cancer patients with a second cancer (2C patients) and carefully matched controls without a second cancer (1C patients). Skin fibroblasts represent a normal somatic cell type. In contrast to blood and EBV-transformed lymphoblasts, which can be more easily obtained, primary fibroblasts constitute a homogenous cell population with intact cell cycle and DNA repair checkpoints. So far there have been only few studies on primary fibroblasts of cancer patients. Fibroblasts of breast and thyroid cancer patients were found to have defective repair and/or cell cycle regulation [13]. Abnormal gene expression in somatic cells of the unaffected parents of retinoblastoma patients are also consistent with an inherited predisposition to cancer development [14].

To identify susceptibility factors for second cancer formation, we screened various DNA-repair associated genes for constitutive protein expression differences in 2C versus 1C patients. The DNA damage checkpoint protein RAD9A was downregulated in both untreated and irradiated somatic cells of two-cancer patients, compared to one-cancer patients. Increased constitutive and DNA damage-induced levels of RAD9A protein and other genomic caretakers may help to maintain genome stability and prevent second tumor development after radiation and chemotherapy. RAD9A, which in some papers is called hRAD9 or simply RAD9, is an interesting candidate, because it functions in multiple pathways, including DNA repair, cell cycle checkpoint control and apoptosis and its abnormal expression has been linked to tumorigenesis [15], [16].

## Results

### Recruitment of patients

Twenty individuals who survived a childhood cancer and then developed a second cancer were recruited from the German Childhood Cancer Registery. At least one year must have passed since diagnosis of the second cancer. Twenty matched cases who survived a childhood cancer and did not develop a second cancer were randomly chosen from the Registery. The matching criteria were same sex, equal primary cancer diagnosis, equal age at first diagnosis, and same time under observation. Because the primary tumors of matched one- and two-cancer patients were diagnosed in the same year, the treatment modalities were largely identical. All patients were followed up from primary cancer diagnosis to the time when they were recruited.

The patients had to be alive and at least 18 years of age (legal age in Germany) to give their informed consent to skin biopsy. Less than 50% of the contacted two-cancer patients decided to participate in the study. Because of these inclusion criteria, we could only recruit a limited number of two-cancer patients throughout Germany. There is a certain bias in the distribution of primary and secondary cancers. For example, the most frequent combination of acute myeloid leukemia after acute lymphoid leukemia has a very unfavourable prognosis and, therefore, is not represented. On the other hand, secondary thyroid carcinomas are overrepresented, because the patients have a good prognosis and reach adulthood.

### Reduced levels of DNA repair-associated proteins in cells of two-cancer patients

**Figure 1:**
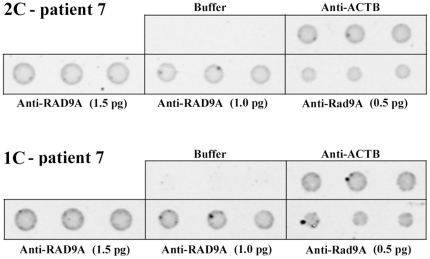
Representative antibody microarray. Different amounts (approximately 1.5, 1.0 and 0.5 pg) of anti-RAD9A antibody (2 ng/µl) are spotted in triplicates onto nitrocellulose-coated slides and incubated with fluorescent-labeled nuclear protein extract of untreated fibroblasts from two-cancer patient 2C-7 and the matched one-cancer patient 1C-7, respectively. Anti-ACTB serves as positive and spotting buffer as negative control. The measured 2C/1C RAD9A protein ratio is 0.5.

Customized antibody microarrays (for example, see Figure 1) were used to compare the constitutive expression levels (without induction of DNA damage) of different DNA repair-associated proteins in exponentially growing primary fibroblasts of childhood cancer patients with and without second tumor, respectively. The 19 studied genes (Table 1) were selected, because they are key players in different DNA repair pathways (i.e. DNA double-strand break repair, nucleotide excision repair, base excision repair, and mismatch repair), participating either in signaling of DNA damage, checkpoint control and/or DNA repair. Mutations in many of these genes are known to predispose to the development of cancer.

**Table 1:** Constitutive expression differences of DNA repair-associated proteins in fibroblasts of 2C vs. 1C patients, measured by antibody microarrays.

Protein	Antibody	2C/1C ratio	p value	Fold change
ACTB (control)	Sigma #A5441	0.99	0.575	−1.01
ATM	Santa Cruz #sc-7230	1.38	0.117	1.38
APEX	Novus Biol. #NB100-116	1.08	0.627	1.08
BRCA1	Santa Cruz #sc-1553	0.74	0.017	−1.36
BRCA2	Santa Cruz #sc-1817	0.90	0.263	−1.11
DDIT3	Santa Cruz #sc-793	0.79	0.011	−1.27
ERCC1	Santa Cruz #sc-71072	1.60	0.247	1.60
GADD45	Santa Cruz #sc-793	0.82	0.153	−1.23
H2AX	Upstate #05-636	1.47	0.100	1.47
Ku86	Santa Cruz #sc-5280	1.23	0.502	1.23
MLH1	Santa Cruz, #sc-582	1.07	0.247	1.07
MSH6	Santa Cruz #sc-1242	0.86	0.021	−1.16
PCNA	Santa Cruz, #sc-56	0.96	0.232	−1.04
PMS1	Santa Cruz #sc-615	0.92	0.709	−1.08
PMS2	Santa Cruz #sc-617	0.99	0.737	−1.01
RAD9A	Abcam #ab13600	0.73	0.040	−1.38
RAD51	Abcam #ab63801	0.73	0.009	−1.37
RCC1	Santa Cruz sc-1162	0.78	0.263	−1.28
TP53	Santa Cruz #sc-100	0.85	0.003	−1.18
XPA	Santa Cruz #sc-853	0.89	0.115	−1.12

**Figure 2:**
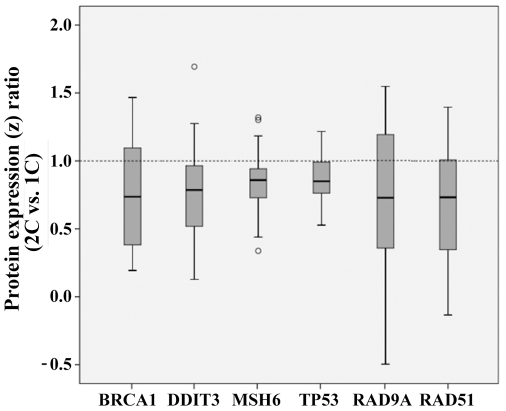
Reduced expression of DNA repair-associated proteins in two-cancer patients. The relative expression levels in fibroblasts of 2C versus 1C patients are −1.36x (p = 0.017) for BRCA1, −1.27x (p = 0.011) for DDIT3, −1.16x (p = 0.021) for MSH6, −1.18x (p = 0.003) for TP53, −1.38x (p = 0.040) for RAD9A, and −1.37 (p = 0.009) for RAD51. Protein expression was measured by antibody microarrays (normalized by log10 transformation and z scores). Box plots show the distribution of z ratios in matched 2C vs. 1C patients. The median is represented by horizontal lines. The bottom of the box indicates the 25^th^ percentile, the top the 75^th^ percentile. The T bars extend from the boxes to at most 1.5 times the height of the box. Outliers are shown as open circles.

**Figure 3:**
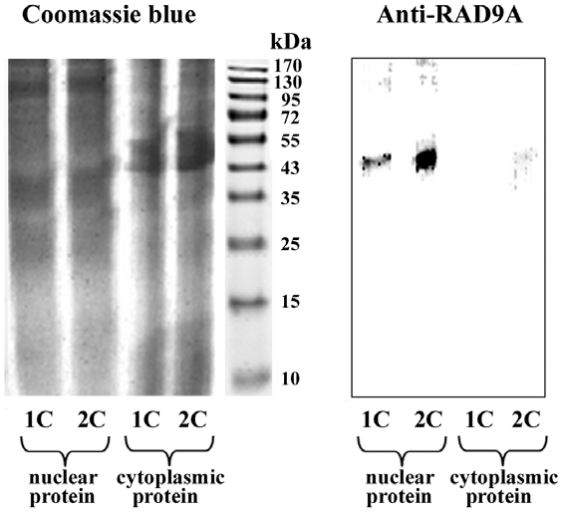
Western blot showing reduced RAD9A protein levels in a two-cancer patient. The gel on the left side shows Coomassie blue staining of nuclear and cytoplasmic protein extracts (30 µg each) from untreated fibroblasts of two-cancer patient 2C-7 and the matched one-cancer patient 1C-7. The corresponding gel on the right side is stained with anti-RAD9A antibody, which recognizes a 45 kDA nuclear protein. The calculated 2C/1C RAD9A protein ratio is 0.6.

For each matched (2C vs. 1C) patient pair we determined the z ratio of triplicate measurements of protein levels (normalized with log10 transformation and z scores). Six of the 19 tested proteins, representing different DNA repair pathways, displayed lower levels in two-cancer patients (Table 1). The box plots in Figure 2 present the distribution of z ratios for BRCA1 (−1.36x; p = 0.017), DDIT3 (−1.27x; p = 0.011), MSH6 (−1.16x; p = 0.021), TP53 (−1.18x; p = 0.003), RAD9A (−1.38x; p = 0.040), and RAD51 (−1.37x; p = 0.009). The p values were not corrected for multiple testing and should be considered as explorative. In order to demonstrate the reliability of our antibody microarrays, Western blot analysis of RAD9A was performed on a representative matched pair. The anti-RAD9A antibody stained the expected 45 kDa band in nuclear protein extracts, whereas no or only a faint band was seen in cytoplasmic extracts (Fig. 3). Consistent with the antibody microarray (Fig. 1), the Western blot showed a lower amount (60%) of RAD9A protein in the 2C patient, compared to the matched 1C patient.

**Figure 4:**
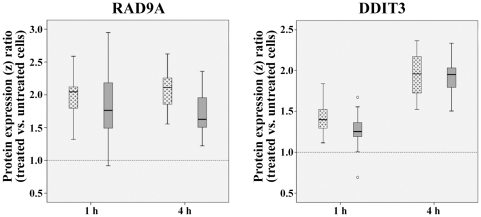
Reduced DNA damage response of two-cancer patients. Differential induction of RAD9A (left side) and DDIT (right side) at 1 h and 4 h after 1 Gy γ-irradiation in fibroblasts of two-cancer patients (gray boxes) and one-cancer patients (dotted boxes). Protein expression was measured by antibody microarrays (normalized by log10 transformation and z scores). Box plots show the distribution of z ratios of treated vs. untreated cells of the same patients. The median is represented by horizontal lines. The bottom of the box indicates the 25^th^ percentile, the top the 75^th^ percentile. The T bars extend from the boxes to at most 1.5 times the height of the box. Outliers are shown as open circles. The DNA-damage induced increase in the 2C group is lower than that in the 1C group for RAD9A at 4 h after irradiation (−1.44x, p = 0.012) and for DDIT3 at 1 h after irradiation (−1.13x, p = 0.019).

The six proteins showing constitutive expression differences were also quantified in cells at 1 h and 4 h after 1 Gy γ-irradiation. For each patient, we compared the protein levels measured by antibody microarrays in treated vs. untreated samples. Two proteins, RAD9A and DDIT3, differed in their cellular response to DNA damage between 2C and 1C patients. The box plots in Figure 4 show the z ratios for RAD9A (after normalization with log10 transformation and z scores) in the 2C and the 1C group. In both groups the RAD9A protein levels were elevated after DNA damage. In the one-cancer group, RAD9A was overexpressed more than twofold at 1 h and 4 h after irradiation, compared to the constitutive protein level. In the 2C group, the amount of RAD9A protein increased 1.76- and 1.63-fold at 1 h and 4 h, respectively, implying a lower induction (−1.44x, p = 0.012 at 4 h) by DNA damage in childhood cancer patients with a second tumor. Similar to RAD9A, the protein encoded by the DNA damage inducible transcript *DDIT3* was also found to be increased in irradiated cells (Fig. 3). In 1C patients, the protein level was elevated 1.40- and 1.96-fold at 1 h and 4 h after DNA damage, compared to 1.26- and 1.95-fold in the 2C group. At 1 h after irradiation there was a lower induction (−1.13x, p = 0.019) in the two-cancer group.

### Reduced RAD9A mRNA expression levels in two-cancer patients

**Figure 5:**
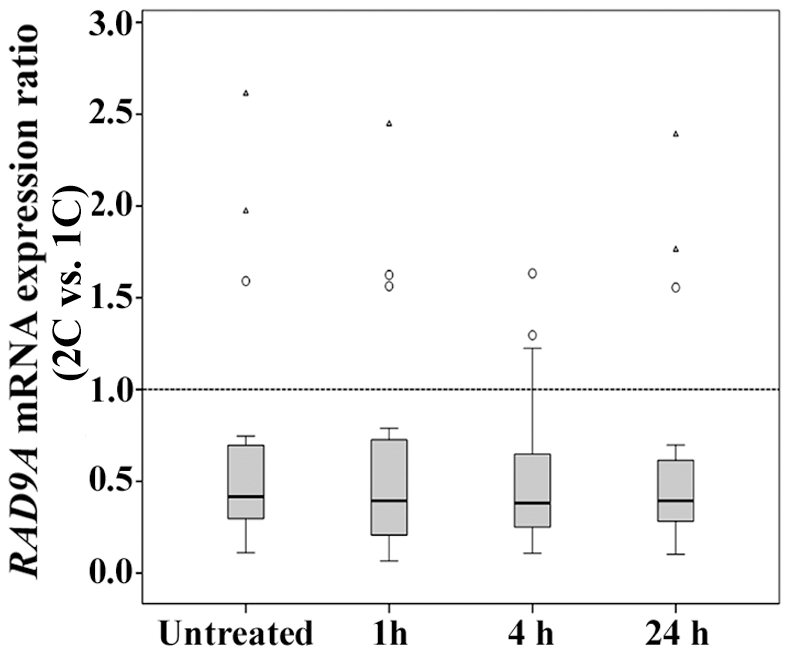
Reduced mRNA expression of *RAD9A* in two-cancer patients. *RAD9A* mRNA levels in untreated and irradiated (1 h, 4 h and 24 h after 1 Gy) fibroblasts of two-cancer patients, compared to matched one-cancer patients. mRNA was quantified by real-time RT PCR (normalized with the ΔΔCT method and two endogenous control genes). Box plots show the distribution of expression ratios in matched 2C vs. 1C patients. The median is represented by horizontal lines. The bottom of the box indicates the 25^th^ percentile, the top the 75^th^ percentile. The T bars extend from the boxes to at most 1.5 times the height of the box. Outliers are shown as open circles, extreme outliers as triangles. Two-cancer patients show reduced *RAD9A* mRNA levels without induction of DNA damage (−2.40x, p = 0.004) as well as at 1 h (−2.54x, p = 0.003), 4 h (−2.62x, p = 0.003), and 24 h (−2.54x, p = 0.003) after irradiation.

Because RAD9A protein was most dramatically downregulated in two-cancer patients, we focussed our further study on this cell cycle checkpoint and DNA repair protein. We performed quantitative mRNA expression analyses by real-time RT PCR in both untreated and irradiated cells. The box plots in Figure 5 present the distribution of expression ratios in 20 matched pairs of patients. The constitutive *RAD9A* mRNA levels (without induction of DNA damage) were significantly lower in two-cancer patients (−2.40x, p = 0.004), compared to one-cancer patients. Between-group differences were also observed at 1 h (−2.54x, p = 0.003), 4 h (−2.62x, p = 0.003), and 24 h (−2.54x, p = 0.003) after irradiation. Three matched pairs represent outliers or extreme outliers in the box plot diagrams, indicating that relative *RAD9A* expression levels can considerably vary between patients and are not always reduced in two-cancer patients. The (extreme) outliers represent different combinations of primary and secondary cancer.

Because it has been reported that *RAD9A* expression is dependent on DNA methylation [17], we determined the methylation status of the presumed cis-regulatory region by bisulfite pyrosequencing. Compared to classic bisulfite plasmid sequencing, bisulfite pyrosequencing can only analyze a limited number of CpG sites located at the most 30–50 bp 3′ from the sequencing primer, however on the other hand it can much more exactly (±2%) quantify the CpG methylation level. The analyzed DNA segment, which contains three adjacent CpG sites, was found to be unmethylated in untreated cells of both 2C and 1C patients. The range of methylation values was 4–10%, as expected for a transcriptionally active gene. There was no significant between-group methylation difference, which could explain the observed expression difference. Because the density of methylated CpGs rather than individual sites in a CpG island turn a gene on or off [18], [19], the average methylation of a few CpGs can usually serve as a representative epigenetic marker for a given cis-regulatory region.

Chromosome 11q13.1 containing the *RAD9A* gene is frequently amplified in a variety of human tumors [17]. To exclude *RAD9A* copy number variations between 2C and 1C patients, we performed high-resolution karyotype analyses with the Affymetrix GeneChip Genome Wide Human SNP array 6.0 as well as quantitative real-time PCR. Both methods revealed two copies of the *RAD9A* gene in all studied patients.

## Discussion

Compared to the enormous efforts to characterize the transcriptomes and proteomes of tumor cells, there are relatively few studies searching for gene expression differences in normal somatic cells of tumor patients [13], [14]. To test the hypothesis that differences in DNA repair pathways may influence the risk for developing a second tumor following treatment of childhood cancer, we compared the constitutive levels of DNA repair-associated proteins in primary fibroblasts of matched two-cancer and one-cancer patients. Because we did not expect dramatic differences but rather subtle modulations in the DNA repair capacity in two-cancer patients, we did not perform a genome-wide screen but tested only a limited number of well-known DNA repair-associated genes using highly sensitive antibody microarrays. The observation that 6 of 19 tested DNA repair-associated proteins were constitutively downregulated in normal cells of two-cancer patients promotes the idea that the DNA repair pathways of two-cancer patients are less capable to handle DNA damage than those of one-cancer patients. For two proteins we also showed a lower induction after DNA damage. One gene, *RAD9A* was analyzed in more detail. Both constitutive mRNA expression in exponentially growing fibroblasts as well as DNA-damage induced expression at different time points after irradiation was lower in two-cancer patients than in one-cancer patients. In this light, RAD9A is a good candidate for a factor predisposing to second cancer. The differential *RAD9A* expression was not mediated by DNA methylation or copy number variation.

The *RAD9A* gene is evolutionarily highly conserved from yeast to man, which is generally considered a good indicator for functional significance. It acts in multiple pathways including base excision, homologous recombination and mismatch repair as well as cell cycle checkpoint control and apoptosis. Many of its functions appear to be mediated by the nuclear RAD9A-HUS1-Rad1 protein complex that resembles PCNA [15], [16]. Mouse *Rad9* ^−*/*−^ and to a lesser extent *Rad9^+/^* ^−^ knockout cells [20] and human RNAi knockdown cells with reduced *RAD9A* levels [21] are sensitive to different types of DNA damage, displaying genome instability, DNA repair deficiency and altered cell cycle checkpoints. Embryonic lethality of the mouse *Rad9* ^−*/*−^ mutation indicates that the multiple functions of Rad9A are essential for embryogenesis and normal development. Mice with targeted *Rad9* ^−*/*−^ deletion in keratinocytes are highly susceptible to genotoxin-induced skin tumor formation [22], [23]. *Rad9* ^−*/*−^ keratinocytes display a higher number of spontaneous and genotoxin-induced DNA breaks, aberrant cell cycle distribution and an increased rate of apoptosis. This is consistent with the view that RAD9A functions as a tumor suppressor in skin and other tissues by promoting DNA repair in damaged cells and stabilizing the genome before tumorigenesis occurs. It is interesting to note that both downregulation and upregulation of *RAD9A* have been associated with tumorigenesis. *RAD9A* overexpression has been observed in a variety of tumors, including breast [17], lung [24], thyroid [25], and prostate cancer [26]. This suggests that *RAD9A* can also function as an oncogene, most likely by aberrant transactivation of downstream target genes. In general, the *RAD9A* level correlated with tumor size and/or stage. *RAD9A* belongs to a growing group of genes with dual roles in cancer. Depending on the cell type and tissue environment, it can demonstrate either tumor-promoting or tumor-suppressing activity [15]. The mechanisms by which the multifunctional RAD9A protein acts as an oncogene and a tumor suppressor, respectively are largely unknown.

Because the number of two-cancer patients being 18 years or older is relatively small, we could not limit our studied patients to a specific tumor entity or treatment protocol. The largest subgroup of primary tumors were lymphoid leukemias (10 cases). Due to their good prognosis, thyroid carcinomas (6 cases) were overrepresented as second cancers. Radiation therapy is an important risk factor for thyroid carcinoma [27]. In this light, it tempting to speculate that the abundance of RAD9A protein may modulate the risk for radiation-induced tumors. Future more comprehensive studies should consider the effects of different treatment modalities of childhood cancer. However, as outlined above, recruiting homogenous groups of patients is extremely challenging. We obtained skin biopsies and blood samples from all our studied patients. Because many patients were bone-marrow transplanted, the blood cells were not analyzed. Fibroblast cultures were established one or more years after second cancer diagnosis/treatment, which makes it unlikely that the expression differences between two-cancer versus one-cancer patients are directly or indirectly influenced by radiation or chemotherapy. Although it is plausible to assume that cultured fibroblasts represent the situation in normal body cells, it would be desirable to also study uncultured cells and/or other tissues.

The number of analyzed patients is relatively small. Nevertheless, our results suggest that the constitutive RAD9A protein levels as well as the extent of induction after DNA damage vary between two-cancer and one-childhood cancer patients. We propose that RAD9A functions as tumor suppressor in skin fibroblasts and other normal cells of the body and, thus, helps to prevent second cancer. Analysis of various normal cell types in larger patient populations, for example adult cancer survivors are necessary to support a role for RAD9A in DNA damage-induced carcinogenesis.

## Materials and Methods

### Patient samples

The German Childhood Cancer Registry has collected almost completely all childhood cancers in Germany since 1980, conducting an open-end follow-up with an emphasis on second neoplasms. With the help of the Registery, we recruited 20 persons who survived a childhood malignancy and then, unrelated to the first event, developed a second cancer (2C patients) as well as 20 carefully matched persons [same sex, same primary cancer (ICCC classification), equal age (±1 year) at first diagnosis] who did not develop a second malignancy (1C patients). Genetic counselling was offered and informed written content was obtained from all patients participating in the study, which was approved by the Ethics Committee of the Medical Association of Rhineland-Palatinate (No. 837.440.03[4102]).

The mean age at diagnosis of the first tumor was 6.8 years (range 0–14) in both groups. The primary tumors were acute myeloid or lymphoid leukemia (11 cases), Hodgkin or Burkitt lymphoma (5 cases) and other solid tumors (4 cases). The mean age at second diagnosis in the 2C group was 16.7 years (range 9–30). The second tumors were myelodysplastic syndrome or lymphoma (7 cases), thyroid carcinoma (6 cases) and other solid tumors (7 cases). Second neoplasms were confirmed by experienced clinical oncologists to be no relapses or alternative manifestations of the primary neoplasm.

Skin biopsies were taken at the earliest one year, usually several years after second cancer therapy. Primary fibroblast cell cultures were established from skin biopsies and cultured in minimal essential medium with Earle’s salts (Invitrogen, Karlsruhe, Germany), supplemented with 10% fetal bovine serum, vitamins and antibiotics. Cells (without DNA damage) were harvested from exponentially growing subconfluent cultures. To induce DNA repair, subconfluent cultures were exposed to 1 Gy ionizing radiation using a GammaCell 2000 (Cs137) irradiator. Samples were taken 1 h, 4 h and 24 h after irradiation. Cells were washed twice with PBS and stored at −80°C until further use.

### Western blot and antibody microarray

For nuclear protein extraction cells were resuspended two times in 500 µl of 10 mM HEPES, 1.5 mM MgCl2, 10 mM KCl, pH 7.9 and incubated on ice for 10 min. After 10 s centrifugation at maximum speed the supernatant was discarded. Then the pellet was resuspended in 100 µl of 20 mM HEPES, 0.42 M NaCl, 1.5 mM MgCl2, 0.2 mM EDTA, 25% glycerol, pH 7.9 and homogenized using a syringe with gauge needle. After incubation on ice for 30 min and centrifugation for 30 min at maximum speed at 4°C, the supernatant containing the nuclear extract was separated from the cytoplasmic pellet and stored at −80°C. The protein concentration was measured according to Bradford, using Roti Quant (Roth, Karlsruhe, Germany).

For Western blot analysis, 30 µg nuclear protein extract were separated on a 8% SDS-PAGE gel and then transferred to a Hybond-P membrane (Amersham, Arlington Heights, IL, USA). The membrane was first blocked with 5% non-fat dry milk (NFDM) dissolved in Tris-buffered saline (50 mM Tris-Cl, pH 7.5, 150 mM NaCl), 0.1% Tween 20 (TBST). The blots were then incubated overnight at 4°C with mouse monoclonal anti-RAD9A antibody, diluted 1∶250 (2 µg/ml) in TBS with 5% NFDM. After washing the blot three times for 5 min with TBST, proteins were detected with peroxidase-labeled secondary rabbit anti-mouse antibodies using the BM Chemiluminescence Western Blotting Kit (Roche Diagnostics, Mannheim, Germany). Band intensities were quantified with a LAS-3000 (Fujifilm, Düsseldorf, Germany) luminescent image analyzer. Comassie blue staining was used to adjust the signal intensities to the amount of protein.

Customized antibody microarrays for quantification of 19 different DNA repair-associated proteins (Table 1) were prepared by spotting one drop, two drops and/or three drops, each drop containing approximately 0.5 pg antibody in triplicates onto nitrocellulose-coated slides (Oncyte, nitrocellulose 16 multi-pad slides, Grace Bio-Labs, Bend, OR, USA), using a non-contact array spotter (sciFLEXARRAYER 3, Scienion, Berlin, Germany). Antibodies against beta-actin (ACTB) served as positive, spotting buffer as negative control. Slides were stored at 4°C in dry condition. Nuclear proteins were labeled with an amine reactive fluorine dye, which forms a covalent amide bond between the primary amines of proteins. Two micrograms of protein and 0.12 µl fluorescent dye (Dylight 649 NHS Ester, Pierce, Rockford, USA) were incubated for 1 h at room temperature in the dark. Then excess fluorescent dye was inactivated by adding 100 mM glycine to the reaction. Prior to use, antibody microarrays were covered with 16-pad FAST frame hybridization chambers (Whatman, Maidstone, UK). Unspecific binding sites were blocked for 1 h at 4°C with 120 µl PBS containing 4% NFDM per subarray, followed by three washes with 120 µl PBS each for 10 min. Labelled protein samples were incubated on sub-arrays overnight at 4°C. Afterwards, the slides were washed two times for 15 min with PBS, 5% Tween 20 and two times for 15 min with HPLC-grade water. Finally, the slides were dried in a SpeedVac and scanned with a high-resolution confocal scanner (Affymetrix array scanner 428 TM, High Wycombe, UK). Slide images were analyzed using the Spotfinder 3.1.1 software (TM4, Dana Faber Cancer Institute, Boston, USA). Background subtraction was performed according to the formula: spot intensity = mean intensity SP - (sum bkg – sum top25 bkg)/(number of pixelSP - number of pixel top25 bkg), where SP represents any spot, bkg the corresponding background and top25 bkg the top 25% of background pixel.

For statistical analysis of microarray data we performed log10 transformation, z score and z ratio calculations [28]. For between-group comparisons we used the sign test and, if possible, the Wilcoxon test (skewness [−1,+1]) and box plots as graphics (PASW statistics 18.0). No adjustment for multiple testing was performed. The analyses were regarded as explorative, and the p values of the corresponding tests are presented for descriptive reasons. The results of these tests can therefore not be considered as significant at any level.

### Quantitative real-time RT PCR

Total RNAs were prepared from treated and untreated fibroblast cultures using the TRIzol method (Invitrogen). One microgram of the RNA samples was reversely transcribed into cDNA using the SuperScript III First-Strand Synthesis System (Invitrogen). Quantitative real-time RT PCR of *RAD9A* was performed with a QuantiTect Primer Assay (Qiagen, Hilden, Germany) and an Applied Biosystems 7500 Fast Real-Time PCR system (Life Technologies, Karlsruhe, Germany). Exon-spanning forward (5′-GAGAAGACGGTGGAAAAATG-3′) and reverse (5′-GGAAGGACAGGTTGTGAGTC-3′) primers were designed with the Primer3, version 0.4.0 (http://frodo.wi.mit.edu/primer3/) program. Linearity of amplification was verified by qPCR standard curve and sequence analysis. *RRN18S* (Qiagen, #QT00199367) and *TBP* (#QT00000721) were used as endogenous control genes. All reactions were performed in triplicates. Each 25 µl reaction volume contained 25 ng cDNA template in 10 µl RNase-free PCR graded water, 2.5 µl 10x QuantiTect Primer Assay and 12.5 µl 2x QuantiTect SYBR Green PCR Master Mix (Qiagen). PCR was performed in two stages with one cycle of 95°C for 15 min (first stage) and 40 cycles of 94°C for 15 s, 55°C for 30 s, and 72°C for 40 s (second stage). Relative quantification was carried out with the ΔΔCT method using the two endogenous control genes and the one-tumor group as calibrator (2C vs. 1C comparison). To quantify protein induction after DNA damage, the untreated cells of each patient were used as calibrator. Group comparisons were performed with the sign test and box plots. No adjustment for multiple testing was performed.

### Methylation analysis

The methylation status of a 900 bp long CpG island ranging from the promoter into intron 2 of the *RAD9A* gene was determined by bisulfite pyrosequencing. The assays targets three representative CpG sites in the second intron of this putative cis-regulatory region [17]. Genomic DNA was isolated with the QIAamp Mini DNA Kit (Qiagen). Bisulfite conversion of 1 µg DNA was performed with the EpiTect Bisulfite Kit (Qiagen) according to the manufacturer’s instructions. *RAD9A* was amplified from bisulfite-converted DNA using forward primer 5′-GGTTTTTATGGGGAAAGGAGG-3′ and biotinylated reverse primer 5′-CCACAAACCCAACCCTCTAAC-3′. Primers were designed with the Pyrosequencing Assay Design Software (Biotage, Uppsala, Sweden). Pyrosequencing was performed with the sequencing primer 5′-TTTTATGGGGAAAGGA-3′ and the PyroGold SQA reagent kit (Qiagen) on a PSQ96MA system (Biotage). Data were analyzed with the Pyro Q-CpG software (Biotage).

### Molecular karyotype analysis

High-resolution screening for microdeletions and duplications was performed with the Affymetrix GeneChip Genome Wide Human SNP array 6.0 and the GeneChip Genome Wide SNP Sty Assay Kit 5.0/6.0, following the protocol developed by the manufacturer (Affymetrix, Santa Clara, CA, USA). Data calculation was performed with Affymetrix Genotyping Console 4.0 and Chromosome Analysis Suite 1.0.1.

Quantitative real-time PCR with QuantiTect SYBR Green-based chemistry was used to validate *RAD9A* copy numbers. PCR was performed with *RAD9A* forward primer 5′-AGGCTGTTCTGCCCTTCTC-3′ and reverse primer 5′-TGCCTCCTCCTCGTGGTA-3′ on an ABI 7500 Fast Real-Time PCR system with one cycle of 95°C for 15 min and 45 cycles of 95°C for 30 s, 60°C for 60 s and 72°C for 40 s. Copy number calculation was performed with the ΔΔCT method, using the *RFC3* gene (forward primer 5′-AGTAGGTGCTTGGCGGTTC-3′, reverse primer 5′-AGTGTAACTTGACCTACATCTTCAATG-3′) as a reference. All experiments were performed in triplicates.
